# Estimation of standardized real-time fatality rate for ongoing epidemics

**DOI:** 10.1371/journal.pone.0303861

**Published:** 2024-05-21

**Authors:** Yuanke Qu, Chun Yin Lee

**Affiliations:** 1 Department of Computer Science and Engineering, Guangdong Ocean University, Zhanjiang, People’s Republic of China; 2 Department of Applied Mathematics, The Hong Kong Polytechnic University, Hong Kong; Fiji National University, FIJI

## Abstract

**Background:**

The fatality rate is a crucial metric for guiding public health policies during an ongoing epidemic. For COVID-19, the age structure of the confirmed cases changes over time, bringing a substantial impact on the real-time estimation of fatality. A ‘spurious decrease’ in fatality rate can be caused by a shift in confirmed cases towards younger ages even if the fatalities remain unchanged across different ages.

**Methods:**

To address this issue, we propose a standardized real-time fatality rate estimator. A simulation study is conducted to evaluate the performance of the estimator. The proposed method is applied for real-time fatality rate estimation of COVID-19 in Germany from March 2020 to May 2022.

**Findings:**

The simulation results suggest that the proposed estimator can provide an accurate trend of disease fatality in all cases, while the existing estimator may convey a misleading signal of the actual situation when the changes in temporal age distribution take place. The application to Germany data shows that there was an increment in the fatality rate at the implementation of the ‘live with COVID’ strategy.

**Conclusions:**

As many countries have chosen to coexist with the coronavirus, frequent examination of the fatality rate is of paramount importance.

## Introduction

As the prospect of herd immunity fades, achieving zero COVID-19 infections appears unfeasible without extremely stringent public health measures, which will come at the cost of a deteriorating economy. After more than two years of combating the COVID-19 pandemic, many parts of the world, including Europe, the United States, and much of Asia, have phased out hard restrictions and returned to normal life since early 2022. While a surge in COVID-19 cases was expected following the reopening of borders and the easing of public health restrictions, the rate of death, hospitalization, and serious illness have remained low in most countries. This suggests that the number of COVID-19 cases is no longer an appropriate metric for determining which and when the control measures are implemented. Health professionals advise against solely relying on the daily number of COVID-19 cases to inform public health policies but to adopt a more comprehensive approach tracking the disease severity, which provides a more accurate picture of disease burden and fatalities, especially in the era of vaccination [1, 2].

The literature on the severity of COVID-19 has primarily focused on the case fatality rate (CFR), which measures the proportion of the number of deaths among confirmed cases up to a specific time, for different populations. Considering that there is a significant time lag between disease onset and death for COVID-19 infection, various methods have been proposed to reduce the bias in the estimation of CFR. These include restricting the analysis to cases with definitive clinical outcomes (died or recovered) or adjusting the value of the cumulative number of cases in the denominator, using the information on the epidemic growth rate and the distribution of time from disease onset to death [3–5].

Nevertheless, estimators developed based on aggregated counts, namely the cumulative numbers of cases and deaths, are generally unable to capture changes in disease fatality over time. In the context of the persisting COVID-19 pandemic involving frequent viral mutations, calculating the overall fatality rate up to a certain point in time may not provide meaningful insights. Instead, continuously monitoring the real-time fatality rate, or in other words, tracking changes in disease fatality rate, is crucial. This metric often yields more valuable information that can inform public health policies, including shifts in disease virulence, the efficacy of response strategies, and the quality of healthcare services. To this end, Qu et al. [6] proposed a real-time fatality rate estimator adjusted for reporting delay (rtaCFR) based on the fused lasso technique. The rtaCFR was shown to outperform other fatality rate estimators in tracking disease severity throughout the COVID-19 pandemic in Germany. They considered the rtaCFR a key metric to guide public health policies as it can capture the changes in disease fatality in real-time, such as a decreasing trend due to the implementation of effective interventions, or an upward trend due to the emergence of a more virulent or vaccine-resistant variant.

However, a limitation of the study is that the effects of explanatory variables, such as age, gender and other demographic variables are not considered. Since the onset of the COVID-19 pandemic in 2020, it has been well-known that the disease outcome of COVID-19 patients varies markedly by age, with older patients facing a higher risk of severe illness and death [7–9]. Therefore, age demographic has long been highlighted as a crucial factor in elucidating the disparities in COVID-19 fatality rates among different countries [10–15]. For instance, India cited its low case fatality rate as evidence of its success in containing the COVID-19 crisis. However, the seemingly low fatality is presumably attributed to its young-skewed population, who are less susceptible to severe disease outcomes [16]. When accounting for age distribution, India could be in a much worse position than other nations. This example illustrates how the population age structure can affect the overall fatality rate. Nevertheless, limited attention has been paid to the fact that the age distribution among confirmed cases also changes throughout the epidemic. Staerk et al. [17] showed that variations in the estimated effective infection fatality rates could be attributed to a shift in the age distribution of confirmed cases towards older age groups. Such an impact could lead to misinterpretations of the real-time fatality rates, and misguided public health policies.

In this article, we propose a standardized real-time adjusted case fatality rate estimator (srtaCFR) that can serve as an indicator for the government to better understand the situation in the midst of a pandemic. The proposed method corrects the bias of the commonly used CFR by adjusting for the time lags between illness onset and death, and is more sensitive in picking up the changes in fatality rate during an ongoing epidemic. A distinguished feature of the new estimator compared to the existing rtaCFR in [6] is its ability to remove the effects of some time-varying confounding factors attributed to the changes in disease severity when the relevant information is available. One typical application of the proposed method is to adjust for the impact induced by the changes in the age distribution of confirmed cases when monitoring changes in disease virulence over time. For instance, at the beginning of the COVID-19 epidemic, the majority of cases were concentrated in the older and more vulnerable population, which partly contributed to the relatively high fatality rate therein. Hence, the overall fatality could be overestimated even if the disease virulence remains unchanged. Similarly, if there is a shift towards younger individuals being diagnosed with COVID-19, it could potentially lead to a spurious decline in disease severity within that period. Therefore, adjusting for the effects of age distribution helps to provide a more accurate picture of COVID-19, and is crucial in informing policy decisions. Simulation studies demonstrate the superiority of the proposed srtaCFR in terms of high accuracy and sensitivity in capturing the changes in disease severity throughout the epidemic. Under the influence of the temporal age distribution, the proposed method is shown to be empirically unbiased in all scenarios, whereas the existing rtaCFR performs well only when the underlying age-specific fatality rates are constant and identical over time; otherwise, rtaCFR may result in a delayed or inaccurate representation of the trends in disease severity. We illustrate the usefulness of the proposed method through an application to the COVID-19 data in Germany from March 2020 to May 2022.

## Methods

### Basic setting

Assume that epidemiological data are collected regularly, such as on a daily basis, during an emerging epidemic. Let *τ* be the current time point or the end of an epidemic. Throughout the article, we always label a time point in the subscript of a notation, if any. For *t* = 1, …, *τ*, the basic data collected at time *t* includes the number of confirmed cases *c*_*t*_ and the number of reported deaths *d*_*t*_. It is assumed that each confirmed case will eventually die or recover. For infectious diseases, there will be a significant time lag between illness onset and death. Let *F* denote the cumulative distribution function of the time from disease onset to death, which can be informed by some prior knowledge, such as estimates obtained from previous outbreaks or from the analysis of some hospitalized cases where individual-level data are available. Given an outcome of death, we assume that the death is reported *s* days after the time of diagnosis with probability *f*_*s*_ = *F*_*s*_ − *F*_*s*−1_, the difference of *F* evaluated at time *s* and *s* − 1 respectively.

### Real-time fatality rate estimator

A real-time fatality rate estimator taking into account reporting delay in death has been recently proposed by Qu et al. [6], where minimal epidemiological data, namely the observed time series of *c*_*t*_ and *d*_*t*_, and the distribution *F*, are required as inputs. We will briefly mention the existing estimator to facilitate the discussion of its standardized version. Let *p*_*t*_ denote the (unknown) probability that a confirmed case reported on day *t* will eventually die from the disease, for *t* = 1, …, *τ*. Clearly, the expected total number of deaths at time *t* ignoring reporting delay is given by *c*_*t*_*p*_*t*_. In the presence of reporting delay, these *c*_*t*_*p*_*t*_ individuals will die subsequently with a delay distributed according to *F*. In this model, the expected number of deaths at time *t*, which can be regarded as a function of (*p*_1_, …, *p*_*t*_), is given by

$$\begin{array}{c} d_{t}^{*}=\sum_{s=0}^{t-1}p_{t-s}c_{t-s}\;f_{s+1},\;t=1,\ldots ,\tau . \end{array}$$ (1)

Let $d^{*}={\left(d_{1}^{*},\ldots ,d_{\tau }^{*}\right)}^{T}$, ***c*** = (*c*_1_, …, *c*_*τ*_)^*T*^ and ***p*** = (*p*_1_, …, *p*_*τ*_)^*T*^. By considering the temporal structure of the data, d* can be expressed in matrix form ***Qp*** where

$$\begin{array}{c} Q=(\begin{array}{cccc} f_{1} & 0 & \ldots & 0\\ f_{2} & f_{1} & \ldots & 0\\ \vdots & & \ddots & \vdots \\ f_{\tau } & \ldots & f_{2} & f_{1} \end{array})(\begin{array}{cccc} c_{1} & 0 & \ldots & 0\\ 0 & c_{2} & & \vdots \\ \vdots & & \ddots & 0\\ 0 & \ldots & 0 & c_{\tau } \end{array}) \end{array}$$

is a deterministic matrix which can be constructed by ***c*** and *F*. Thus, the estimation of ***p*** can be simplified to a linear regression problem. By setting p* = *p*_1_ = ⋯ = *p*_*τ*_ in (1), the above framework is reduced to that of the time-delay adjusted case fatality rate estimator studied in Nishiura et al. [18], denoted as p* in this article. Given the strong serial correlation in the time series of *p*_*t*_ and the fact that typically only the trend of the time series is of interest, Qu et al. [6] proposed to apply the fused lasso technique [19, 20] to obtain a smoothed estimator of ***p***, namely the real-time delay-adjusted case fatality rate, henceforth rtaCFR_*t*_ for *t* = 1, …, *τ*. Let ***d*** = (*d*_1_, …, *d*_*τ*_)^*T*^ and

$$\begin{array}{c} D=(\begin{array}{ccccc} 1 & -1 & 0 & \ldots & 0\\ 0 & 1 & -1 & \ldots & 0\\ \vdots & & \ddots & \ddots & \vdots \\ 0 & \ldots & 0 & 1 & -1 \end{array}) \end{array}$$

be a (*τ* − 1) × *τ* penalty matrix. That is to solve the following minimization problem

$$\begin{array}{c} \text{rtaCFR}=\hat{p}=\underset{p}{\text{argmin}}\;\frac{1}{2}{∥d-Qp∥}_{2}^{2}+\lambda {∥Dp∥}_{1}, \end{array}$$ (2)

where **rtaCFR** = (rtaCFR_1_, …, rtaCFR_*τ*_)^*T*^, λ > 0 is a trade-off parameter that weights the accuracy of the solution and its sparsity level. As λ increases, the adjacent coordinates in $\hat{p}$ are pooled towards each other, which results in smoother estimates of real-time fatality rates. Given the expression in (2), the solution path of the generalized lasso problem can be computed using the R package genlasso [21] where the residual sum of squares value associated with each pre-assigned λ value can be obtained. In real data analysis, we can first consider the candidates in the solution path which satisfies $0\leq {\hat{p}}_{t}\leq 1$ for all *t*, then choose λ = λ* that yields the smallest residual sum of squares.

### Standardized real-time fatality rate estimator

The aforementioned rtaCFR estimator is derived based on the minimal data requirements, without considering the effects of explanatory variables such as age, sex and some healthcare burden indicators are not considered. It is a composite index that takes into account a basket of time-varying indistinguishable factors when interpreting trends of the fatality rate over time. In other words, the effects of various underlying factors are mixed together, and we cannot segregate the effect of one factor from that of others. Consequently, the effect of a factor of interest may be masked by the effect of another less important confounding factor. For instance, it is widely accepted that the fatality rate of a COVID-19 patient increases with age. If the effectiveness of a certain public health policy is evaluated based on a composite index that does not account for age, the actual effect of a successful policy may be obscured or confounded by the increasing proportion of elderly individuals among the confirmed cases over the period of policy implementation, thus providing misleading signals to the policymakers. This raises the question of whether a standardized version of rtaCFR can be developed when additional information on a confounding variable is collected along with the minimal epidemiological data.

Suppose that an additional individual-level variable with *J* (*J* > 1) categories, denoted by *X*, is available throughout the observation period. Then, for *j* = 1, …, *J*, the stratified epidemiological data may be represented by ***c***^(1)^, …, ***c***^(*J*)^ and ***d***^(1)^, …, ***d***^(*J*)^, where $c^{\left(j\right)}={\left(c_{1}^{\left(j\right)},\ldots ,c_{\tau }^{\left(j\right)}\right)}^{T}$ and $d^{\left(j\right)}={\left(d_{1}^{\left(j\right)},\ldots ,d_{\tau }^{\left(j\right)}\right)}^{T}$ denote the time series of confirmed cases and deaths in the *j*th category, respectively. Furthermore, let $p^{\left(j\right)}={\left(p_{1}^{\left(j\right)},\ldots ,p_{\tau }^{\left(j\right)}\right)}^{T}$ be the fatality rates for group *j*. Based on the formulation in (2), a group-specific real-time fatality rate estimator, denoted as **rtaCFR**^(*j*)^, of ***p***^(*j*)^ can be obtained for the *j*th category based on the stratified data ***c***^(*j*)^, ***d***^(*j*)^ and *F*, for *j* = 1, …, *J*. Thus, a standardized real-time delay-adjusted case fatality rate estimator is given by

$$\begin{array}{c} {\text{srtaCFR}}_{t}=\sum_{j=1}^{J}q_{t}^{\left(j\right)}{\text{rtaCFR}}_{t}^{\left(j\right)},\;\text{for}\;t=1,\ldots ,\tau , \end{array}$$ (3)

where $q_{t}^{\left(j\right)}$ denotes a deterministic, possibly time-dependent reference weight for confirmed cases belonging to the *j*th category at time *t*, such that $\sum_{j=1}^{J}q_{t}^{\left(j\right)}=1$ for all *t*. Similar to (2), we define **srtaCFR** = (srtaCFR_1_, …, srtaCFR_*τ*_)^*T*^. It follows that one can easily remove the temporal effects of *X* on the real-time fatality rates by assigning a time-independent sequence (*q*^(1)^, …, *q*^(*J*)^) in (3). For instance, to control for the effects on fatality rates induced by a shift in the age distribution of the confirmed cases (where *X* represents the age group), one can assign (*q*^(1)^, …, *q*^(*J*)^) to be the time-independent population age structure at time 0 as the reference distribution. In this case, srtaCFR pertains to the weighted average of the age-specific real-time fatality rates for the reference distribution, at which the infections are assumed to always occur randomly and uniformly over the population throughout the observation period.

## Simulation studies

We investigate the empirical properties of the proposed srtaCFR estimator in contrast to the existing rtaCFR estimator under different hypothetical scenarios, to provide some insights into why the former metric would be more appropriate for assessing the severity of an infectious disease in real-time. Consider a scenario where minimal epidemiological data is available over a period of *τ* = 200 intervals (e.g. in days). This data consists of the observed time series of *c*_*t*_ and *d*_*t*_ for *t* = 1, …, 200, while the exact infection and death times of each individual are not obtainable. Furthermore, the collected time series data can be stratified based on a demographic variable *X*. This mimics the real-world situation of most epidemic outbreaks, where detailed individual-level data may not be readily accessible, as an infected subject may not be followed up regularly as in the clinical setups. However, demographic information such as age, gender or health conditions may still be accessible. To facilitate interpretation, we can consider treating *X* as the age variable, as age is a well-known influential factor in COVID-19 fatality. Specifically, the number of confirmed cases at each time point *t* can be stratified into *J* = 3 broad age groups, namely (1) young, (2) middle, and (3) old age groups.

**Fig 1:**
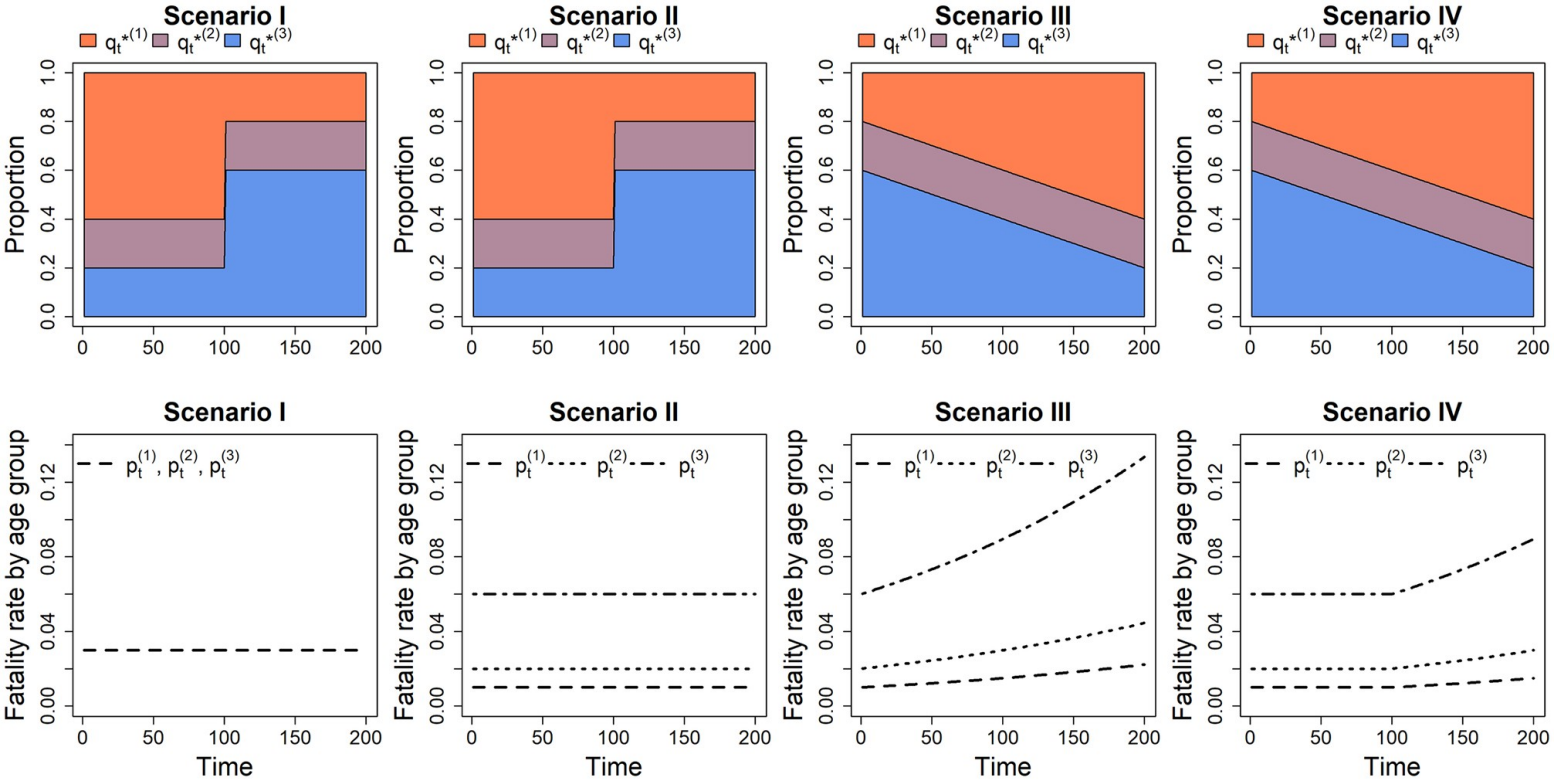
Simulation settings in scenarios I–IV. Top panel: temporal age distributions of confirmed cases, where $q_{t}^{*(1)}$, $q_{t}^{*(2)}$, and $q_{t}^{*(3)}$ represent the proportion of young, middle and old age groups, respectively. Bottom panel: patterns of the temporal age-specific fatality rates, where $p_{t}^{\left(1\right)}$, $p_{t}^{\left(2\right)}$ and $p_{t}^{\left(3\right)}$ represent the fatality rates of the young, middle and old age groups, respectively.

Let $q_{t}^{*(1)},q_{t}^{*(2)}$ and $q_{t}^{*(3)}$ be the underlying proportions of confirmed cases in young, middle and old age groups at time *t*, respectively, for *t* = 1, …, 200. We consider four typical scenarios, where the temporal age distributions, namely ${\left\{q_{t}^{*(1)},q_{t}^{*(2)},q_{t}^{*(3)}\right\}}_{t=1,\ldots ,200}$, and the age-specific fatality rates, namely ${\left\{p_{t}^{\left(1\right)},p_{t}^{\left(2\right)},p_{t}^{\left(3\right)}\right\}}_{t=1,\ldots ,200}$ are depicted in Fig 1. Specifically, we set the proportion of the middle age group to be constant at $q_{t}^{*(2)}=0.2$ for all *t* in all scenarios. In scenarios I and II, the proportion of elderly cases switches from $q_{t}^{*(3)}=0.2$ to $q_{t}^{*(3)}=0.6$ at *t* = 100. This shift mimics the real-world situation that the emergence of a novel variant tends to have a greater impact on more vulnerable populations. In scenarios III and IV, we assume a shift towards younger age groups by setting a linearly decreasing proportion of elderly cases, $q_{t}^{*(3)}=0.6-0.002t$. This pertains to the situation where the disease is spreading more and more rapidly among younger individuals, possibly due to various factors, such as changes in population behaviour or increased susceptibility among younger age groups. Regarding the fatality rates, we set $p_{t}^{\left(1\right)}=p_{t}^{\left(2\right)}=p_{t}^{\left(3\right)}=0.03$ in scenario I, and $p_{t}^{\left(1\right)}=0.1,p_{t}^{\left(2\right)}=0.02,p_{t}^{\left(3\right)}=0.06$ in scenario II. In scenario III, the fatality rates increase exponentially over the entire period, with $p_{t}^{\left(1\right)}=0.1\;\text{exp}\;\left(0.004t\right)$, $p_{t}^{\left(2\right)}=0.02\;\text{exp}\;\left(0.004t\right)$, and $p_{t}^{\left(3\right)}=0.06\;\text{exp}\;\left(0.004t\right)$. In scenario IV, the fatality rates are set to be $p_{t}^{\left(1\right)}=0.1\;\text{exp}\;\left\{0.004{\left(t-100\right)}_{+}\right\},p_{t}^{\left(2\right)}=0.02\;\text{exp}\;\left\{0.004{\left(t-100\right)}_{+}\right\},p_{t}^{\left(3\right)}=0.06\;\text{exp}\left\{0.004{\left(t-100\right)}_{+}\right\}$, where *a*_+_ = *a* if the constant *a* > 0, and *a*_+_ = 0 otherwise. In summary, the age-specific fatality rates remain constant and identical in scenario I, constant but different in scenario II, while in scenario III, they increase exponentially throughout the observation period. In scenario IV, the fatality rates remain constant initially but begin to increase exponentially after *t* > 100, indicating the emergence of a more virulent mutant variant.

The following briefly describes the simulation setup. The non-stratified number of confirmed cases is set to *c*_*t*_ = 10000 − 50 ⋅ |100 − *t*| for *t* = 1, …, 200. This mimics the real-world situation where a surge in confirmed cases is typically observed in a new wave of infection, followed by a decline resulting from the implementation of some effective control measures. Given *c*_*t*_ at time *t*, we then generate the age-stratified confirmed cases $\left(c_{t}^{\left(1\right)},c_{t}^{\left(2\right)},c_{t}^{\left(3\right)}\right)$ based on the multinomial distribution with probabilities $\left(q_{t}^{*(1)},q_{t}^{*(2)},q_{t}^{*(3)}\right)$ provided in the top panel of Fig 1. For a particular group *j*, the total number of deaths contributed by $c_{t}^{\left(j\right)}$ is generated based on the binomial distribution with probability $p_{t}^{\left(j\right)}$ provided in the bottom panel of Fig 1. The death figures will be reported (realized) in future time points (*t* + *t*_*delay*_), where *t*_*delay*_ is distributed according to *F* to capture the delay between the onset of disease and death. We assume that *F* follows a gamma distribution with a mean of *μ* = 15.43 days and shape parameter *γ* = 2.03, referring to the situation of COVID-19 outbreak estimated by recent studies [22]. Therefore, we obtain $d^{\left(j\right)}={\left(d_{1}^{\left(j\right)},\ldots ,d_{\tau }^{\left(j\right)}\right)}^{T}$ by summing up the reported deaths at each time point *t* for groups *j* = 1, 2, 3. The total number of reported deaths at time *t* under this case is simply given by $d_{t}=\sum_{j=1}^{J}d_{t}^{\left(j\right)}$.

**Fig 2:**
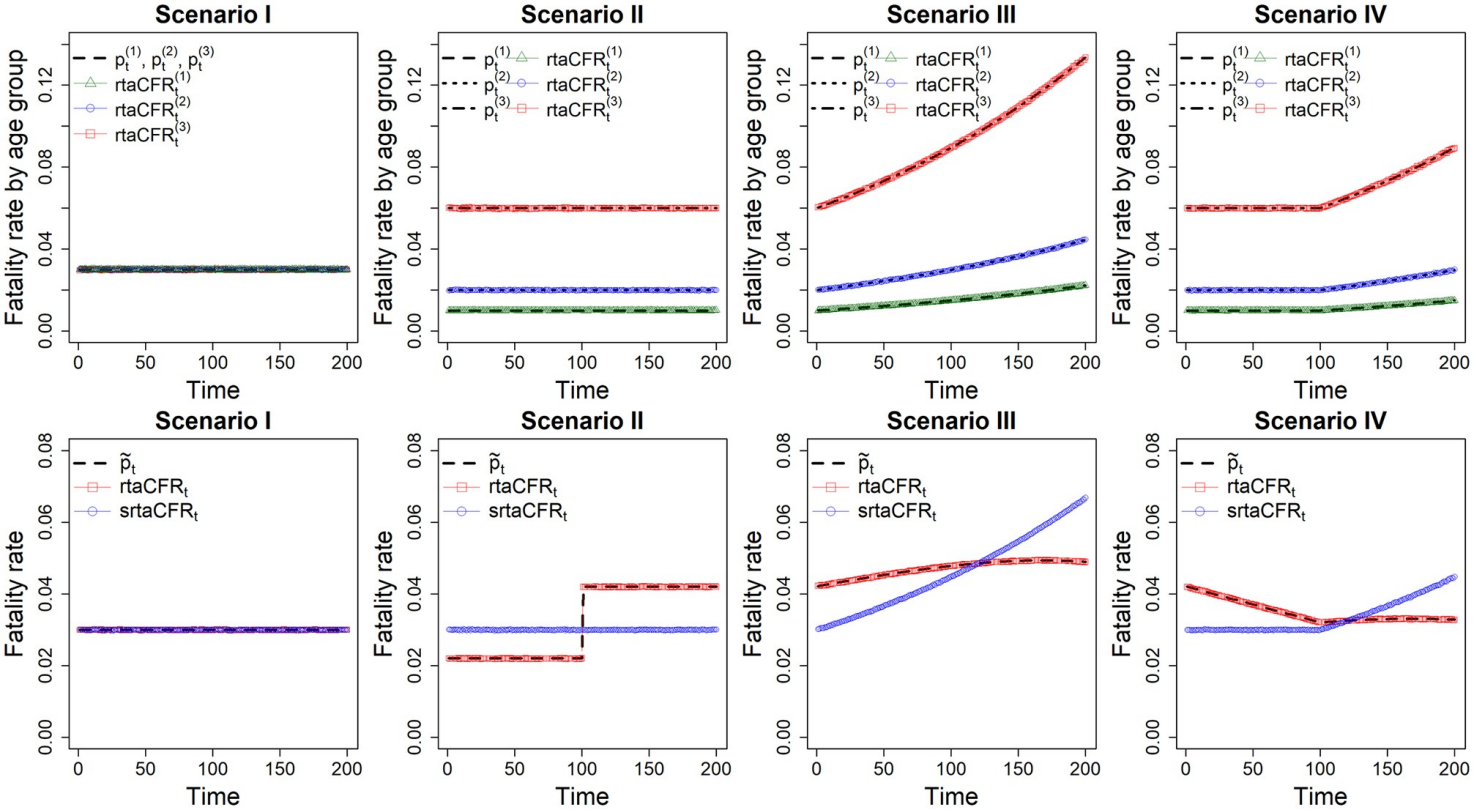
Simulation results under scenarios I–IV. Top panel: empirical averages of the age-specific real-time fatality estimates ${\text{rtaCFR}}_{t}^{\left(j\right)},j=1,2,3$, overlayed with the true age-specific fatality rates. Bottom panel: the underlying overall fatality rate $\tilde{p}$, and empirical averages of the estimates rtaCFR_*t*_ and srtaCFR_*t*_.

We consider 1000 replications of the above simulation procedure. For each replication, we compute the age-specific real-time fatality rate **rtaCFR**^(*j*)^ for each of the three age groups (*j* = 1, 2, 3) based on the simulated age-stratified confirmed cases ***c***^(*j*)^ and deaths ***d***^(*j*)^. Then, we compute the proposed standardized estimator **srtaCFR** given in (3) based on the time-invariant reference age distribution (*q*^(1)^, *q*^(2)^, *q*^(3)^) = (1/3, 1/3, 1/3), which pertains to the situation that infection occurs uniformly across different age groups. According to (3), **srtaCFR** estimates the weighted averaged fatality rates ${\bar{p}}_{t}=\sum_{j=1}^{3}q^{\left(j\right)}p_{t}^{\left(j\right)}=\sum_{j=1}^{3}p_{t}^{\left(j\right)}/3$, allowing us to comprehensively capture the changing trend of the three pre-specified fatality rates $p_{t}^{\left(j\right)}$’s depicted in Fig 1. In contrast, the **rtaCFR** is computed based on the total (non-stratified) cases ***c*** and deaths ***d***, which is a composite index measuring the overall real-time fatality rates $\tilde{p}=\left({\tilde{p}}_{1},\ldots ,{\tilde{p}}_{\tau }\right)$ where ${\tilde{p}}_{t}=\sum_{j=1}^{3}q_{t}^{*(j)}p_{t}^{\left(j\right)}$ for *t* = 1, …, *τ*. The average estimates of ${\text{rtaCFR}}_{t}^{\left(j\right)}$, rtaCFR_*t*_ and srtaCFR_*t*_ over the 1000 replications, along with ${\tilde{p}}_{t}$ are provided in Fig 2.

As shown in the top panel of Fig 2, the estimated curves for the age-specific real-time fatality rates, ${\text{rtaCFR}}_{t}^{\left(j\right)}$, align closely with their respective true values across all scenarios, showing that the age-specific estimators are unbiased. We then evaluate the performance of the composite estimator, rtaCFR, and the standardized estimator, srtaCFR, across different scenarios. In scenario I where the age-specific fatality rates are constant and identical across all age groups, the temporal age distribution of confirmed cases has no effect on the estimates of disease fatality. We can see in the bottom panel of Fig 2 that rtaCFR_*t*_ and srtaCFR_*t*_ are almost identical and stay at the same level across the time axis in scenario I. However, in scenario II, where the age-specific fatality rates are constant with $p_{t}^{\left(1\right)}<p_{t}^{\left(2\right)}<p_{t}^{\left(3\right)}$, a sudden shift in the age distribution towards the elderly (i.e., a sudden increase in q*^(3)^) at *t* = 100 will result in an increase in the overall fatality rate, ${\tilde{p}}_{t}$, even though the age-specific fatalities remain unchanged throughout the observation period. As expected, the composite estimator rtaCFR_*t*_ aligns closely with ${\tilde{p}}_{t}$, indicating an increase in disease virulence. On the contrary, the proposed standardized fatality rate estimator srtaCFR_*t*_ is totally unaffected by the shift in the age distribution and stays at a constant level because $p_{t}^{\left(1\right)},p_{t}^{\left(2\right)},p_{t}^{\left(3\right)}$ are constants, and the computation of estimator relies on a time-invariant reference age distribution.

In scenario III, where the age distribution gradually shifts towards the youth, and all the age-specific fatality rates are increasing exponentially, the rtaCFR_*t*_ (and also ${\tilde{p}}_{t}$) is almost flat, as the increase in age-specific fatality rates is offset by the shift in the age distribution. However, the standardized fatality rate estimator srtaCFR_*t*_ is increasing exponentially over time, reflecting the actual increase in age-specific fatality rates. Scenario IV is similar to scenario III in terms of the shifting age distribution, but the exponential increase in age-specific fatalities occurs only after *t* > 100. Consequently, the effects of an increase in age-specific fatalities on the overall fatality rate, ${\tilde{p}}_{t}$, are offset or even surpassed by the effects of the skewed young confirmed cases. This leads to a conflicting phenomenon where the non-standardized fatality rate estimator, rtaCFR_*t*_, is decreasing, but the standardized fatality rate estimator, srtaCFR_*t*_, is increasing.

The simulation results highlight the importance of accounting for the age distribution when estimating real-time fatality rates. The impact of the temporal age distribution on the estimates of the fatality rate also depends on the underlying fatality rates for each age group. If the fatality rates are identical across age groups, as in scenario I, both rtaCFR and srtaCFR estimators perform well in capturing the disease virulence in a timely manner. However, it is important to note that this scenario is unlikely to occur in the context of infectious disease, as the fatality rate typically increases with age. In situations where the fatality rates differ across age groups, as seen in scenarios II to IV, the changing age distribution can have a significant impact on the estimation of disease fatality. As demonstrated in scenario II, a sudden shift in the age distribution towards the elderly can induce a false signal of increasing virulence of the disease. This is because the rtaCFR is a weighted average of the age-specific fatality rates, where the weights are determined by the age distribution of confirmed cases. When the age distribution shifts towards the elderly, the fatality rate will increase, even if the underlying age-specific fatality rates remain constant over time. We can see analogous results in scenarios III and IV, where the increase in fatality rate is masked or even surpassed by the decreasing age of confirmed cases.

In real-world applications, policymakers are often interested in estimating the virulence of a disease or evaluating the effectiveness of a control measure, while the impact of changes in age distribution on overall fatality rates is typically considered a nuisance factor. Consequently, the composite index rtaCFR that includes a basket of unknown factors may not be desirable in fatality estimation provided that information on demographic variables, such as the patient’s age, is available. In this simulation study, we provide a very basic example to illustrate that the proposed age-standardized fatality rate estimator can be more appropriate and accurate in reflecting the actual changes in disease fatality, by removing the confounding effects of the age variable. Therefore, the government can have a better evaluation of the true trajectory of the fatality rates promptly when informing public health policies. The real-world epidemiological data structure is far more complex than that used in our simulation settings. Presumably, we may also consider a standardized fatality rate estimator with data stratified on some important factors other than age, given the availability of those data.

### The COVID-19 pandemic in Germany

**Fig 3:**
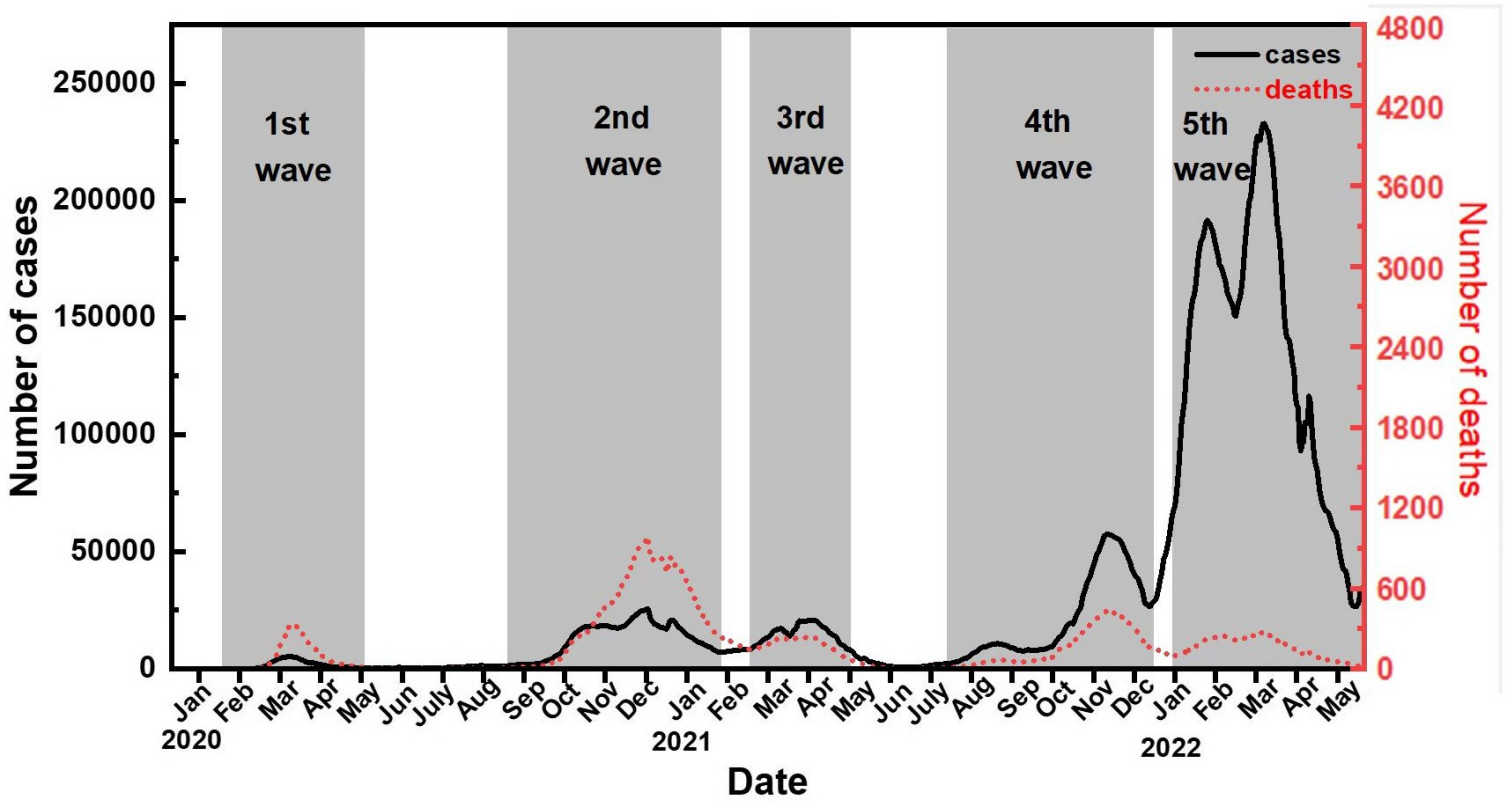
The daily numbers of confirmed cases and deaths in Germany.

The COVID-19 pandemic, caused by the novel coronavirus SARS-CoV-2, emerged in late 2019 in Wuhan, China [23]. Then it spread rapidly across the world, causing sustained outbreaks in early 2020. On March 11, 2020, the World Health Organization declared the COVID-19 outbreak a global pandemic [24]. By the end of March 2020, more than 750,000 cases with around 37,000 deaths have been reported in 200 countries and territories, with Europe and the Americas becoming the new epicentre [25]. Compared to other European nations, Germany was considered a role model that handled the COVID-19 pandemic relatively well during its early stages. The daily numbers of confirmed cases and deaths in Germany with age demographics were collected from a public demographic database [26]. Given that the fatality rate remained at a very low level since the second half of 2022 with the emergence of a milder Omicron subvariant, we aim to examine changes in disease fatality in Germany prior to this period. Fig 3 shows the seven-day moving averages of daily confirmed cases and deaths from March 2020 to May 2022.

The COVID-19 pandemic reached Germany in late February 2020, resulting in a notable surge in the number of cases and deaths. The government reacted quickly, and a range of measures including widespread testing, social distancing, school closure and travel bans have been introduced since early March [17, 27, 28]. Despite these efforts, the first wave of the pandemic hit Germany hard, particularly in the older population due to several outbreaks originating from nursing homes and religious events [28]. The infection reached its peak in early April 2020, with the country reporting over 100,000 cases and more than 2,000 deaths related to COVID-19. Owing to strict measures put in place, especially the nationwide lockdown introduced in late March 2020, Germany was able to successfully reduce the number of COVID-19 infections in the subsequent weeks [29]. By the summer of 2020, cases had significantly decreased, leading to a loosening of restrictions starting in May 2020. However, we can see from Fig 3 that Germany experienced a new surge in COVID-19 cases after the summer holidays. The increased travel demand and social gatherings during the holiday seasons led to a record-high infection within communities in the fall of 2020. In response, the government reintroduced stricter measures in November 2020, including social distancing, closing non-essential businesses and enforcing mandatory mask-wearing requirements in public places [30]. Concurrently, the vaccination campaign in Germany was initiated at the end of 2020 with priority given to the residents of nursing homes and health workers who were at higher risk of severe illness or exposure to the virus [31].

While the situation improved towards the end of 2020, Germany experienced a third wave of the pandemic in early 2021 due to the emergence of the more transmissible Alpha variant, initially identified in the United Kingdom. In response, Germany implemented various restrictive measures and intensified its vaccination campaign, prioritizing older adults and individuals with underlying health conditions. By May 2021, there was an evident decline in the number of cases and hospitalizations since the peak in late March 2021. With infection rates remaining low and an increased vaccination rate among citizens, the government introduced a five-step plan in July 2021 to gradually lift restrictions on businesses and public life. This approach aimed to strike a balance between controlling the spread of the virus and promoting long-term economic growth. It reflected an adaptation to living with COVID after a period of stringent measures [32, 33].

However, the emergence of the Delta variant in the summer of 2021 led to a fourth wave of the pandemic. The relaxed “live with COVID” policy in July was believed to have contributed to the worsening situation. Towards the end of 2021, the already strained healthcare system faced considerable pressure with the rise of the highly contagious Omicron variant, resulting in a large number of infections [34]. As shown in Fig 3, the number of cases increased, with daily counts surpassing 200,000 by mid-February 2022. Notably, COVID-19-related deaths remained relatively low during the fifth wave, attributed to high vaccination rates among the population, particularly among the elderly. As of September 2022, over 90% of individuals aged 60 and older have been fully vaccinated [35].

**Fig 4:**
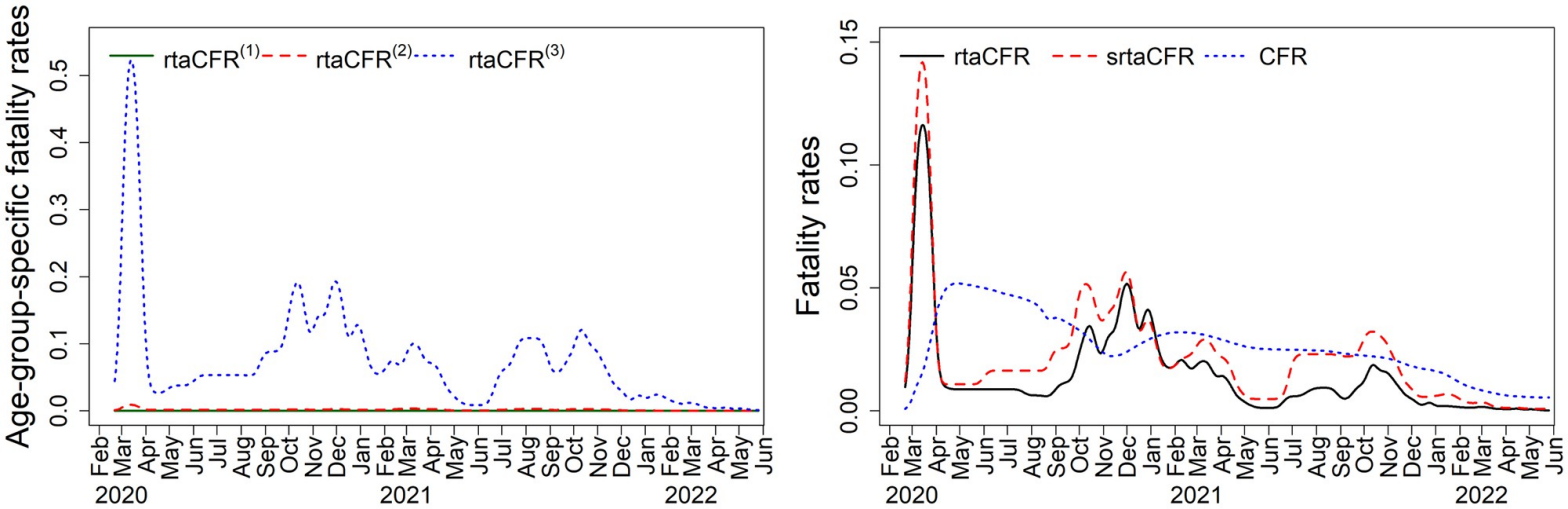
Estimation of the real-time fatality rates for the COVID-19 data in Germany. Left panel: Age-specific fatality rates for groups (1) 0 − 20 years old, (2) 20 − 60 years old, and (3) 60+ years old. Right panel: traditional CFR, rtaCFR_*t*_ and srtaCFR_*t*_.

**Fig 5:**
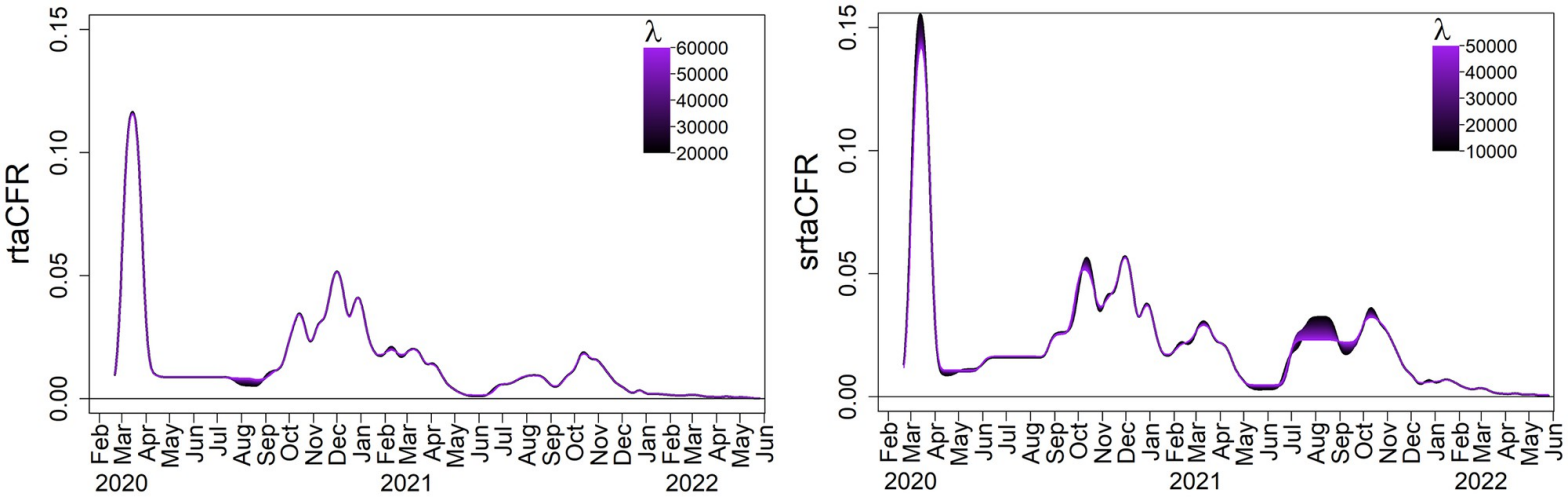
Sensitivity analysis.

We apply **rtaCFR** in (2) and **srtaCFR** in (3) to the COVID-19 data in Germany from March 2020 to May 2022, with an observation period of *τ* = 833 days. The tunning parameter λ* obtained in **rtaCFR** is 28654, while the λ* values for **rtaCFR**^(1)^, **rtaCFR**^(2)^, and **rtaCFR**^(3)^ are 9625, 21771 and 8220, respectively. We assume that *F* follows a gamma distribution with a mean of 15.43 days and a shape parameter of 2.03 [22]. To gain an insight into the trend of fatality rate over time, we apply a Gaussian kernel smoother to the fatality rate estimators with bandwidth 15 days. The left panel of Fig 4 plots the smoothed age-specific fatality rates (i.e. ${\text{rtaCFR}}_{t}^{\left(j\right)}$ for *j* = 1, 2, 3) for ages 0-20, 20-60, and 60+ years. Meanwhile, the red dashed line in the right panel of Fig 4 shows the srtaCFR_*t*_, which is the weighted average of ${\text{rtaCFR}}_{t}^{\left(j\right)}$ with time-independent weights (*q*^(1)^, *q*^(2)^, *q*^(3)^) = (0.20, 0.51, 0.29) corresponding to the population age distribution of Germany as of December 31, 2021. For comparison, we also included the rtaCFR_*t*_ as a black solid line and the traditional CFR as a blue dotted line on the same graph. To investigate the effect of the penalty term λ* in the fused lasso regression, Fig 5 presents a sensitivity analysis showing the smoothed rtaCFR_*t*_ and srtaCFR_*t*_ obtained using a range of different values for λ*. As expected, when λ* increases, a larger penalty is imposed and the neighbouring estimates are getting closer to each other across the time axis, yielding a smoother estimated curve for the real-time fatality rate. The sensitivity analysis demonstrates that the proposed method is robust against the changes in λ*, as the estimated trends of the fatality rates are shown to be insensitive to the selection of λ*.

As illustrated in Fig 4, the CFR increased significantly during the first wave of infection, reaching its peak in May 2020, and it dropped gradually to a low level in 2022 since the emergence of the Omicron variant. While the CFR provides a useful measure of overall disease severity, it is important to recognize that this measure has limitations in capturing the actual variability of the fatality rate in real-time, as it is computed based on cumulative epidemiological figures. As remarked in Qu et al. [6], the CFR is better served as a measure of the severity of a disease with a stable fatality rate spanning a short time frame. On the contrary, both rtaCFR and srtaCFR estimators are competent to capture the changes in disease fatality during the ongoing epidemic, with four peaks of fatality rates attained in April 2020, December 2020, April 2021, and November 2021, respectively. This finding aligns closely with the progression of the first four pandemic waves in Fig 3.

**Fig 6:**
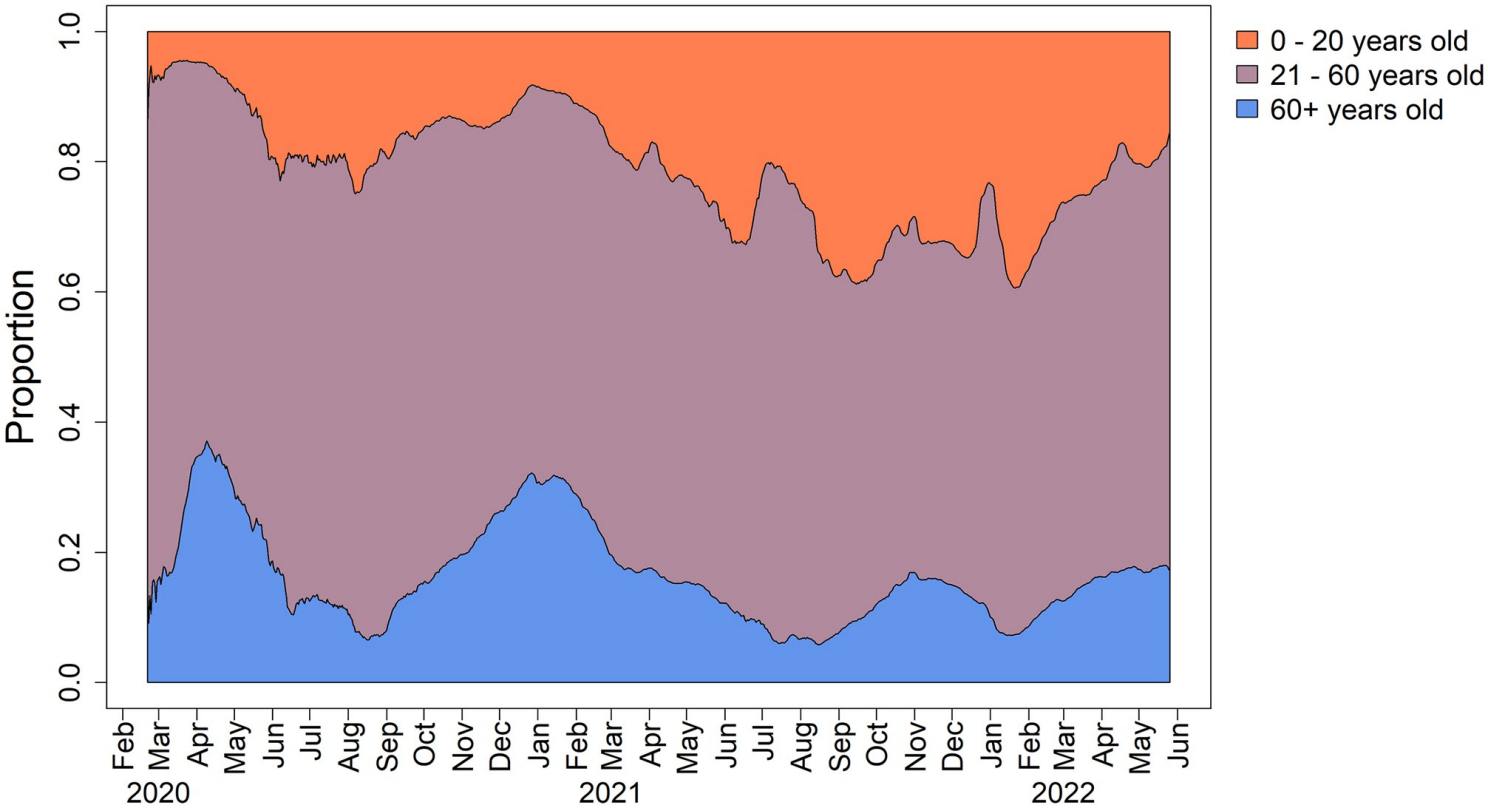
Temporal age distribution of the confirmed cases in Germany.

The estimated trends provided by rtaCFR_*t*_ and srtaCFR_*t*_ are largely similar within the observation period. Nevertheless, there was a noticeable difference between the rtaCFR_*t*_ and srtaCFR_*t*_ during the summer of 2020, with the latter being higher. This difference can be attributed to the fact that the age structure of confirmed cases during the summer of 2020 was different from that of the baseline period. The rtaCFR estimator does not account for changes in age distribution over time, while the srtaCFR estimator adjusts for such changes. The departure is evident based on Fig 6 that the proportion of confirmed cases among young people increased quickly during that period. This shift was possibly induced by the lifting of lockdown measures and the reopening of schools and universities, which led to more social activities among younger people. A similar shift towards young confirmed cases was seen in the summer of 2021 after the government had decided to implement the “live with COVID” policy. The observed pattern is analogous to scenario III in the simulation study, where the actual increasing trend of the fatalities is masked by a younger structure of confirmed cases when one considers rtaCFR.

The “live with COVID” strategy has been widely recognized as a great attempt to return to normalcy, as most of the first countries that adopted this approach in 2021 have observed a stabilization or even decline in COVID-related fatalities while simultaneously rebooting their economies [36]. However, the success of this strategy in improving the case fatality rate may be an illusion due to bias in the estimation of CFR. As demonstrated by the right panel of Fig 4, the implementation of the “live with COVID” policy in June 2021 was associated with a spike in both rtaCFR and srtaCFR estimators. In particular, srtaCFR_*t*_ has risen to a value twice as much as rtaCFR_*t*_ in the second half of 2021. The observed false decreasing trend of the CFR after the implementation of the “live with COVID” policy suggests that the widely-used CFR estimator may not be an appropriate metric for informing policies. Relying on the CFR alone to assess the impact of public health measures may lead to a false sense of security and complacency among the population and policymakers. For example, if the CFR appears to be decreasing, policymakers may assume that the situation is improving and ease public health measures prematurely, potentially leading to a resurgence of the virus. It is also important to note that the discrepancy between the rtaCFR_*t*_ and srtaCFR_*t*_ depends on the changing age distribution of confirmed cases, as well as the pattern of the underlying fatality rates for each age group. Specifically, the greater the shift in the age structure of the confirmed cases, the higher the age-specific fatalities, and the greater the difference between these two measures. Due to the relatively weak virulence of the mutant virus in mid-2021, the difference between the two real-time estimates we observed is not very large. However, the difference between these two estimators would be more pronounced for infectious diseases with a high fatality. As we observed in scenario IV of the simulation study, they may even give the opposite conclusion for changes in disease fatality. Therefore, it is better to consider srtaCFR which accounts for the changing age distribution of confirmed cases to provide a more accurate and reliable picture of the disease’s virulence for tracking the disease’s impact on the population.

## Discussion

Age is a crucial factor contributing to the likelihood of severe illness and death of a patient with COVID-19. This phenomenon is evident when we refer to the estimated age-specific fatality rates in Germany. The fatality for the elderly group is considerably higher than that of the middle and young age groups, with the latter ones being relatively flat and almost zero throughout the pandemic. Hence, the age distribution among confirmed cases can have a substantial impact on the estimation of disease fatality. It is important to account for the changing age distribution of cases, particularly when the government monitors disease fatality to inform public health policy. As demonstrated in the simulation study, a shift in the age structure of cases can potentially result in a spurious decline in the overall disease fatality, even if the age-specific fatalities are unchanged or increasing. The inaccurate evaluation of the fatality trend may lead to delayed or even misguided control measures. To address this issue, we propose a novel standardized real-time fatality rate estimator that corrects bias caused by reporting delay in the traditional CFR and, on the other hand, considers both the changing age structure of confirmed cases and the corresponding age-specific fatalities to provide an accurate estimate of the true disease fatality during an ongoing epidemic.

The application to German data demonstrates the satisfactory performance of the proposed method for its capability to capture the moving trends of the disease fatality, accurately revealing peaks of fatality during each of the four waves of infections. By accounting for the age distribution of confirmed cases, the proposed srtaCFR estimator is capable of reflecting the true increase in fatality rate after the implementation of the “live with COVID” policy in the summer of 2021, while the traditional CFR and the rtaCFR estimator suggest a decreasing or flat trend of disease fatality. It is noteworthy that the pattern of age-specific fatality of COVID-19 cases is rather simple in the application as the estimated trend of overall fatality is dominated by the estimated trend of fatality in the elderly group (aged above 60) since the changes in the fatality rates for both young and middle age groups are negligible. The proposed method can also be applied to other diseases with more complicated patterns of age-specific fatality rates. A typical example is the Ebola virus disease, where the overall case fatality ranges from 30% to 90%, depending on the species of Ebola virus and the quality of medical care provided [37]. Unlike the SARS-CoV-2 coronavirus, all age-specific fatality rates are well above zero for the Ebola virus, with particularly high risk in children aged below 5 and adults aged above 45 [38]. Therefore, the age distribution of the confirmed cases can have a substantial influence on the interpretation of the overall fatality over time. In addition, there is a delay ranging from 6 to 16 days from symptom onset to death for Ebola infections [39]. Hence, the proposed srtaCFR is expected to work well for the estimation of the real-time fatality rate of the Ebola virus disease, adjusting for both reporting delay and structural change in age over time.

To better understand the impact of infectious diseases, it is important to recognize that the fatality rate is a multifaceted measure that can be influenced by factors beyond age. For instance, sex, race, and vaccination status can play important roles, as certain diseases can disproportionately affect patients with different combinations of these variables. Therefore, it is crucial to take these factors into account when assessing the impact of infectious diseases over time. Depending on the characteristics of the infectious disease of interest and the availability of information on relevant factors, the proposed method can be easily extended to accommodate the adjustment for various time-varying nuisance factors based on the multi-level stratified epidemiological data.

A potential avenue for future research is to account for the possible ascertainment bias in estimating the fatality rate. Specifically, the proposed srtaCFR estimator may encounter such bias if the fatality rate of individuals diagnosed with COVID-19 differs from that of individuals who are infected but remain undiagnosed. The extent of this bias is contingent upon the level of test coverage specific to each country. In countries with widespread testing, such as China and Germany, this bias is negligible. However, in countries with less comprehensive testing, such as Italy and the United States, the proposed method may tend to reflect the situation for patients with moderate to severe symptoms, potentially leading to an overestimation of the fatality rates. In these circumstances, additional data about the testing volume and other relevant information is required to estimate the ratio of under-reporting rate [40]. Therefore, there is an urgent need for standardized data collection by national health authorities. Furthermore, in periods of high infection numbers, it is also important to consider the age-specific testing patterns and their potential influence on the estimated real-time fatality rate. If testing capacities are primarily directed towards older individuals who are at a higher risk of severe outcomes, the observed fatality rate may be inflated. This bias should be taken into account when estimating the fatality rate during such periods.

An alternative research approach involves employing indirect standardization, which entails standardizing with the age distribution from a reference population instead of the population of interest adopted by direct standardization. Direct standardization used in our method is known for its high precision, while the indirect alternative proves useful when there is limited availability of detailed age distribution data, as it still yields valuable insights despite potential precision limitations [41, 42].

In light of the federal structure of Germany, it is important to acknowledge the potential spatial variations in epidemiological reporting. Incorporating spatial information into our inference or standardization methods can provide a more comprehensive understanding of the disease dynamics across different regions [43]. As demonstrated in many previous studies [41, 44, 45], such approaches allow us to account for spatial heterogeneity and better capture the nuances of the epidemiological situation. By considering spatial factors, we can enhance the precision and reliability of our analyses, leading to more informed decision-making processes.

In summary, the proposed method provides a more accurate and reliable depiction of the disease’s virulence compared to other existing fatality rate estimators. By monitoring the latest trend in the fatality rate, the continuous decision-making process can be supported during the epidemic. As many countries have chosen to coexist with the coronavirus, frequent examination of the fatality rate is of paramount importance as policymakers can be well-informed when there is a rebound in the fatality of the disease, such that restrictive measures can be implemented promptly.
